# The Identification of Specific Methylation Patterns across Different Cancers

**DOI:** 10.1371/journal.pone.0120361

**Published:** 2015-03-16

**Authors:** Chunlong Zhang, Hongyan Zhao, Jie Li, Hongbo Liu, Fang Wang, Yanjun Wei, Jianzhong Su, Dongwei Zhang, Tiefu Liu, Yan Zhang

**Affiliations:** 1 College of Bioinformatics Science and Technology, Harbin Medical University, Harbin, China; 2 Department of Gastroenterology, The fourth Affiliated Hospital of Harbin Medical University, Harbin, China; 3 Department of General Surgery, The Second Affiliated Hospital of Harbin Medical University, Harbin, China; Sapporo Medical University, JAPAN

## Abstract

Abnormal DNA methylation is known as playing an important role in the tumorgenesis. It is helpful for distinguishing the specificity of diagnosis and therapeutic targets for cancers based on characteristics of DNA methylation patterns across cancers. High throughput DNA methylation analysis provides the possibility to comprehensively filter the epigenetics diversity across various cancers. We integrated whole-genome methylation data detected in 798 samples from seven cancers. The hierarchical clustering revealed the existence of cancer-specific methylation pattern. Then we identified 331 differentially methylated genes across these cancers, most of which (266) were specifically differential methylation in unique cancer. A DNA methylation correlation network (DMCN) was built based on the methylation correlation between these genes. It was shown the hubs in the DMCN were inclined to cancer-specific genes in seven cancers. Further survival analysis using the part of genes in the DMCN revealed high-risk group and low-risk group were distinguished by seven biomarkers (*PCDHB15, WBSCR17, IGF1, GYPC, CYGB, ACTG2*, and *PRRT1*) in breast cancer and eight biomarkers (*ZBTB32, OR51B4, CCL8, TMEFF2, SALL3, GPSM1, MAGEA8*, and *SALL1*) in colon cancer, respectively. At last, a protein-protein interaction network was introduced to verify the biological function of differentially methylated genes. It was shown that *MAP3K14, PTN, ACVR1* and *HCK* sharing different DNA methylation and gene expression across cancers were relatively high degree distribution in PPI network. The study suggested that not only the identified cancer-specific genes provided reference for individual treatment but also the relationship across cancers could be explained by differential DNA methylation.

## Background

Epigenetics is referred to a heritable mechanism that affects the gene expression which is unchanged in DNA sequence [1]. DNA methylation is one of the most important epigenetic events in mammals, and mainly refers to the covalent addition of a methyl group (CH_3_) on the 5’ position of cytosine. A CpG-rich region is called a CpG island which always overlaps on gene promoters and associates with negative regulation of the gene expression [2,3]. It has been revealed that CpG methylation plays an important role in biological processes including imprinting, retrotransposon silencing, X chromatin inactivation, DNA duplication, transcription, repair, even development of cancers and many other complex diseases[4]. Recent researches demonstrate that the methylation mechanism has strong effects within the cancer genes [5,6]. Therefore further understanding of the CpG methylation on complex diseases will be helpful to disease diagnosis, treatment and prognosis. To date, there are an increasing number of studies mainly focusing on mechanism of epigenetic processes, especially CpG methylation [7].

Cancers have been regarded as complex diseases [8]. There are many factors involved in cancers such as copy number alteration, differential expression, epigenetic aberration and so on, among which abnormal DNA methylation has been discovered in many cancers, including breast cancer, lung cancer and colorectal cancer. Hypermethylation in promoter regions inhibits the expression of tumor suppressor genes. On the other hand, hypomethylation in promoter regions activates the oncogene expression. Global hypomethylation also plays an important feature in the process of carcinogenesis, increasing genomic and chromosomal instability [9,10]. For example, many reports have demonstrated that breast cancer is the most widespread cancer in women around the world and has more than six significant subtypes described by gene expression. *BRCA1/2* are the most cancer-related genes in breast cancer, they involve in DNA repair, regulation of transcriptional activation and apoptosis. The hypermethylation of *BRCA1/2* in promoter regions results in the inactivation of function and increases the risk of breast cancer [11,12]. Similarly, the hypermethylation of *AOX1* (aldehyde oxidase 1) and *GSTP1* (Glutathione S-transferase 1) in prostate cancer also lead to the silence of gene expression [13,14]. *LINE-1* (long interspersed nucleotide element-1) is often regarded as a surrogate marker for global DNA methylation and the overexpression of *LINE-1* induced by hypomethylation in promoters results in a poor prognosis in non-small cell lung cancer [15].

A large number of cancer-related genes have been recognized and many significant genes are associated with more than one cancer. *CDH1* (E-cadherin), an intercellular adhesion molecule in epithelial cells plays an important role in establishing and maintaining intercellular connections. The hypermethylation of *CDH1* in colorectal carcinogenesis reduces the gene expression and destroys the function of cell-cell adhesion system [16]. Moreover the hypermethylation of *CDH1* also disrupts the intercellular adhesion in gastric cancer, breast cancer and bladder cancer [17–19]. *RASSF1* (RAS association domain family gene 1) also has high frequency of methylation in promoter regions and acts as a risk factor in prostate cancer and squamous cervical cancer [20–22]. Therefore, it is necessary to develop an integrated analysis across tumors to discover similarities of different cancers and tumor-specific characteristics.

In this study, we constructed an integrated compendium of DNA methylation data across seven cancers. The data was selected from the Cancer Genome Atlas (TCGA) project, including more than 12,000 tumor samples from about 20 tumor types, which provided an opportunity to find gene aberration among different cancers[23]. The comparison of methylation profiles across different samples was used to investigate cancer-related methylation variation and cancer-specific methylation. We quantified the correlation between promoter DNA methylation and gene expression to present the recognition of a DNA methylation cancer-related signature. In addition, it was also shown the association across the cancers through the DNA methylation correlation network(DMCN) constructed in this study. We further reported that the cancer-related biomarker genes could also make a contribution to prognosis through survival analysis in the breast cancer and colon cancer.

## Materials and Methods

### DNA methylation and expression data

DNA methylation data was obtained from the Cancer Genome Atlas (TCGA). The samples consisted of 832 cancer samples and 284 normal samples collected from matched adjacent normal tissues, including breast invasive carcinoma (BRCA), colon adenocarcinoma (COAD), kidney renal clear cell carcinoma (KIRC), kidney renal papillary cell carcinoma (KIRP), lung adenocarcinoma (LUAD), lung squamous cell carcinoma (LUSC), rectum adenocarcinoma (READ) and their matched samples as normal ones. The data was detected by the Infinium HumanMethylation27 BeadChip containing of 27,578 CpG sites in 14,475 genes. The data was from level 2 and β value was defined as DNA methylation level which was calculated as radio of methylated probe (M/ (U+M)) ranging from 0 to 1.

The probes with detection “NA” were treated as missing data. Samples with more than 150 missing probes were treated as missing data and 66 samples were deleted. Next, the sites with missing data were deleted. Coefficient of variation (CV) was used to assess the discrete degree of all data. The genes with CV less than 0.5 in more than 80% samples were remained. CV = SD/AVE. CV was the coefficient of variation of sites belonging the same gene in one sample, SD was the variance of sites, AVE was the average of sites. Average methylation level of multiple CpGs on the same gene was defined as the methylation level of gene.

The expression data was obtained from 210 cancer samples and 68 normal samples (including matched and normal samples) in TCGA. COAD and READ were detected by the platform AgilentG4502A including 17,814 genes. BRCA, KIRC, KIRP, LUAD and LUSC were detected by the platform illminaHiseq_RNASeqV2 including 20,531 genes. The sample information of both DNA methylation data and expression data were listed in S1 Table.

### Identification of differential genes

In this study, we used the R library package “samr” based on t-test statistical significance to identify the differentially methylated genes (DMGs). DMG was defined as significance level false discovery rate (FDR) less than 0.05 and difference of DNA methylation level more than 0.15 between cancers and normal samples [24]. The version of package tool we used was 4.0. In order to understand the mechanism of DNA methylation in cancers, we also used samr to identify the differentially expressed genes (DEGs) with significance level false discovery rate (FDR) less than 0.05.

### Construction of DNA methylation correlation network

The intersection of DMGs and DEGs were used as the nodes in constructing DNA methylation correlation network. Then we calculated the correlation coefficients by the methylation levels of differential genes as the edges, the absolute value of Pearson coefficient correlation was no less than 0.7 with significance level p-value less than 0.05[25].

The network was visualized through the Cytoscape (http://www.cytoscape.org/), an open source software for constructing biological networks. Then, random network was generated through permutation of seven cancer labels for each gene to assess the reliability of DMCN. The data was perturbed in one gene for 1000 times and we calculated the correlation coefficients of the random data to build the random network.

### Survival analysis of differentially methylated genes

The survival analysis was performed based on the prognostic index (PI) to generate the risk groups.

$$\text{PI}=\beta_{\text{1}}{\text{x}}_{\text{1}}+\beta_{\text{2}}{\text{x}}_{\text{2}}+\ldots \ldots +\beta_{\text{p}}{\text{x}}_{\text{p}}$$

where x_p_ was the expression of biomarker genes and *β_p_* was calculated through the COX regression. With the PI increased, the patients would have a poor prognosis. We used the PI to separate the samples into two groups with the same samples to test whether the biomarkers were associated with the survival stage.

### Construction of protein-protein interaction network

We chose an integrated PPI network as background, which was integrated from the Biomolecular Interaction Network Database (BIND), the Biological General Repository for Interaction Data sets (BioGRID), the Database of Interacting Proteins (DIP), the Human Protein Reference Database (HPRD), IntAct, the Molecular IN Teraction database (MINT), the mammalian PPI database of the Munich Information Center on Protein Sequences (MIPS), PDZBase (a PPI database for PDZ-domains) and Reactome. The constructed seed gene set were SIN_D, DOU_D and TRI_D identified above. They were mapped into integrated PPI network and a sub-network was extracted. The sub-network was composed of the seed genes and the genes connecting with seed genes in integrated PPI network. The degree of node in sub-network was defined as the number of genes which were connected with seed genes. It could be used to predict the importance of identified cancer-related DMGs.

## Results

### Differential methylation patterns across cancers

**Fig 1:**
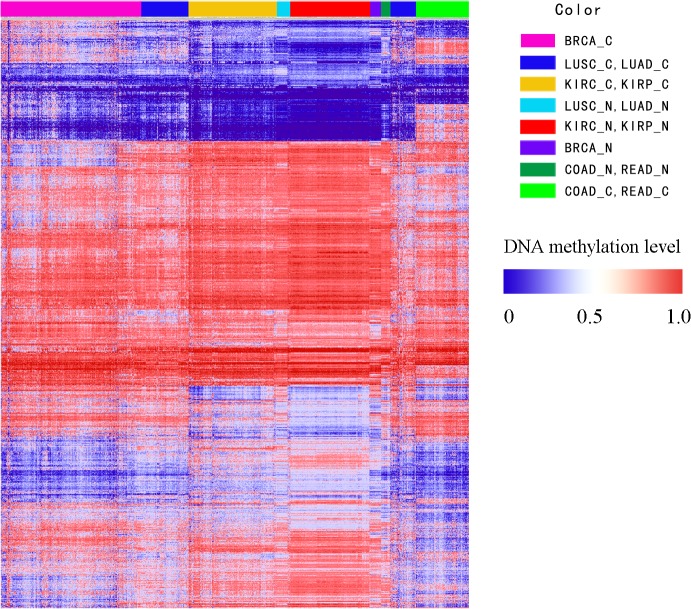
The bidirectional hierarchical clustering of cancer samples of seven phenotypes The letter C represented cancer, and N represented normal. Red was hypermethylation, white is midmethylation and blue was hypomethylation. The samples coming from the same or similar tissues were clustering together, the result of cluster was labeling on the top of the figure.

**Fig 2:**
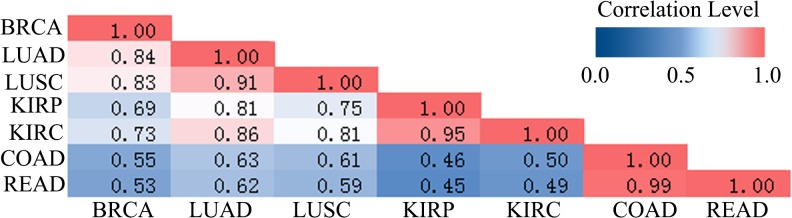
The correlation of seven cancers The pink stood for high correlation coefficient, yellow stood for middle correlation coefficient and green stood for low correlation coefficient. BRCA, LUSC and READ had the strong correlation. Besides this KIRC and KIRP, COAD and READ also had the similar result separately.

Seven cancer data from TCGA were used in this study, including their DNA methylation profile and gene expression profile. After data preprocessing (see materials and methods), 832 cancer samples and 284 normal samples of the seven cancers were remained (Table 1). For the further identification of DMGs, we maintained 7,936 genes whose DNA methylation value existed in all cancers (S2 Table). The DMGs for seven cancers were identified respectively through samr. There were 509 genes showing differential methylation in BRCA, 591 genes in COAD, 130 genes in KIRC, 67 genes in KIRP, 53 genes in LUAD, 508 genes in LUSC and 701 genes in READ (S3 Table). The union of the DMGs was 1,318 (S4 Table). The distribution of methylation patterns of DMGs among cancers were observed based on bidirectional hierarchical clustering analysis (Fig. 1). Samples were clustered into clades based on the specific types of biological samples. The COAD and READ were clustered together which showed similar methylation patterns between these two cancers. Most of DMGs showed high methylation level in COAD and READ compared to other five cancer samples and normal samples, and about 11% in the DMGs displayed specific hypermethylation in COAD and READ respectively. The result was supported that the tumors seemed to be hypermethylated more frequently and there were no significant differences which could distinguish colon and rectum cancers at methylation level [26]. The similar results were also showed in LUAD and LUSC, KIRP and KIRC. To testify the observed results, we calculated the correlation coefficients that displayed the same consequence as hierarchical clustering analysis (Fig. 2). Interestingly, BRCA was high correlated with LUAD and LUSC. They might be caused by the radiotherapy of breast cancer which increased the mortality rate of lung cancers [27]. Moreover, normal samples of above-mentioned cancers also showed similar methylation patterns. On the other hands, cancers from different original organizations had the differential DNA methylation levels (Fig. 1). It was suggested that DNA methylation patterns were associated with tissue and cancer types.

**Table 1:** The number of samples for the identification of DNA methylation patterns and differentially methylated genes.

	CANCER	NORMAL	TOTAL	GENE
**BRCA**	315	27	342	10368
**COAD**	50	17	67	10275
**KIRC**	193	174	367	9925
**KIRP**	16	5	21	10338
**LUAD**	28	4	32	11535
**LUSC**	126	20	146	10347
**READ**	70	5	75	9933

**Fig 3:**
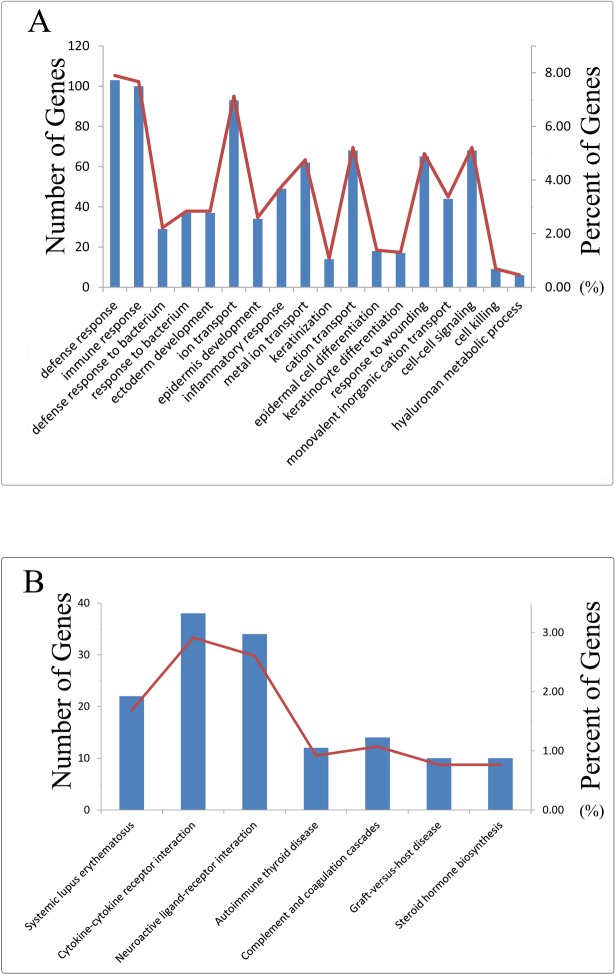
The function enrichment graph of differentially methylated genes The Y axis in the left and the blue histogram stood for the numbers of annotated in KEGG and BP, the Y axis in the right and red curve stood for the percent of annotated genes in BP and KEGG.

Moreover, function enrichment analysis of these 1,318 DMGs was carried using DAVID (http://david.abcc.ncifcrf.gov/) (S5 Table). These genes were mainly enriched in defense response, immune response, cell-cell signaling, cell adhesion, cell killing and so on. Cancer was regarded as a malignant disease and related to defense system, immune response and many other biological processes which involved in unregulated cell growth[28]. Apoptosis was a basic biological process that might have an important relationship with many diseases, such as cancers and autoimmune diseases. Apoptosis was regulated in tumors negatively and the abnormality of apoptosis was involved in cancers [29]. These biological processes might be deregulated by the abnormal methylation of the differential genes, thus affecting the process of cancers. In addition, these genes were associated with many cancer-related pathways (Fig. 3).

### Differentially expressed genes negatively regulated by DNA methylation

DNA methylation in promoter regions had an important function of housekeeping genes. A number of studies indicated that gene expression was regulated negatively by DNA methylation status [30]. In this study, 201 genes of 1,318 DMGs sharing negative relationship between DMGs and DEGs were distinguished. They were divided into 127 hypermethylated genes and 74 hypomethylated genes based on the difference value between cancer samples and normal samples. In further investigation of the function for the negatively related genes, the hypermethylated genes were mainly enriched in cancer-related pathways, including Systemic lupus erythematosus(*CD40*), Cytokine-cytokine receptor interaction(*TNFRSF8, CX3CL1, CD40, IL11RA, ACVR1*), Neuroactive ligand-receptor interaction(*MCHR2, PPYR1, GRIA2*) and autoimmune thyroid disease(*CD40*). Specially, *CD40* was existed in three pathways. CD40 could generate different growth signals between normal tissues and tumors. It was demonstrated to be a tumor suppressor gene and to be hypermethylated in breast cancer (Table 2) [31]. Therefore the methylation of *CD40* supported the evidence for prediction of breast cancer and made an important contribution to the treatment of breast cancer. However hypomethylated genes did not show the clear association with cancer-related pathways. The results suggested that these hypermethylated genes in cancer were cancer-related genes and could be regarded as the candidate biomarkers for the prognosis of cancers.

**Table 2:** The KEGG pathway of the DEGs regulated by DNA methylation.

Category	Name	Hypermethylated	Hypomethylated
**hsa05322**	Systemic lupus erythematosus	CD40	C3,FCGR1A,CD28
**hsa04060**	Cytokine-cytokine receptor interaction	TNFRSF8,CX3CL1,CD40,IL11RA,ACVR1	IL18,CXCL9
**hsa04080**	Neuroactive ligand-receptor interaction	MCHR2,PPYR1,GRIA2	-
**hsa05320**	Autoimmune thyroid disease	CD40	CD28
**hsa04610**	Complement and coagulation cascades	-	C3,F7

### Differential DNA methylation gene correlation network

**Fig 4:**
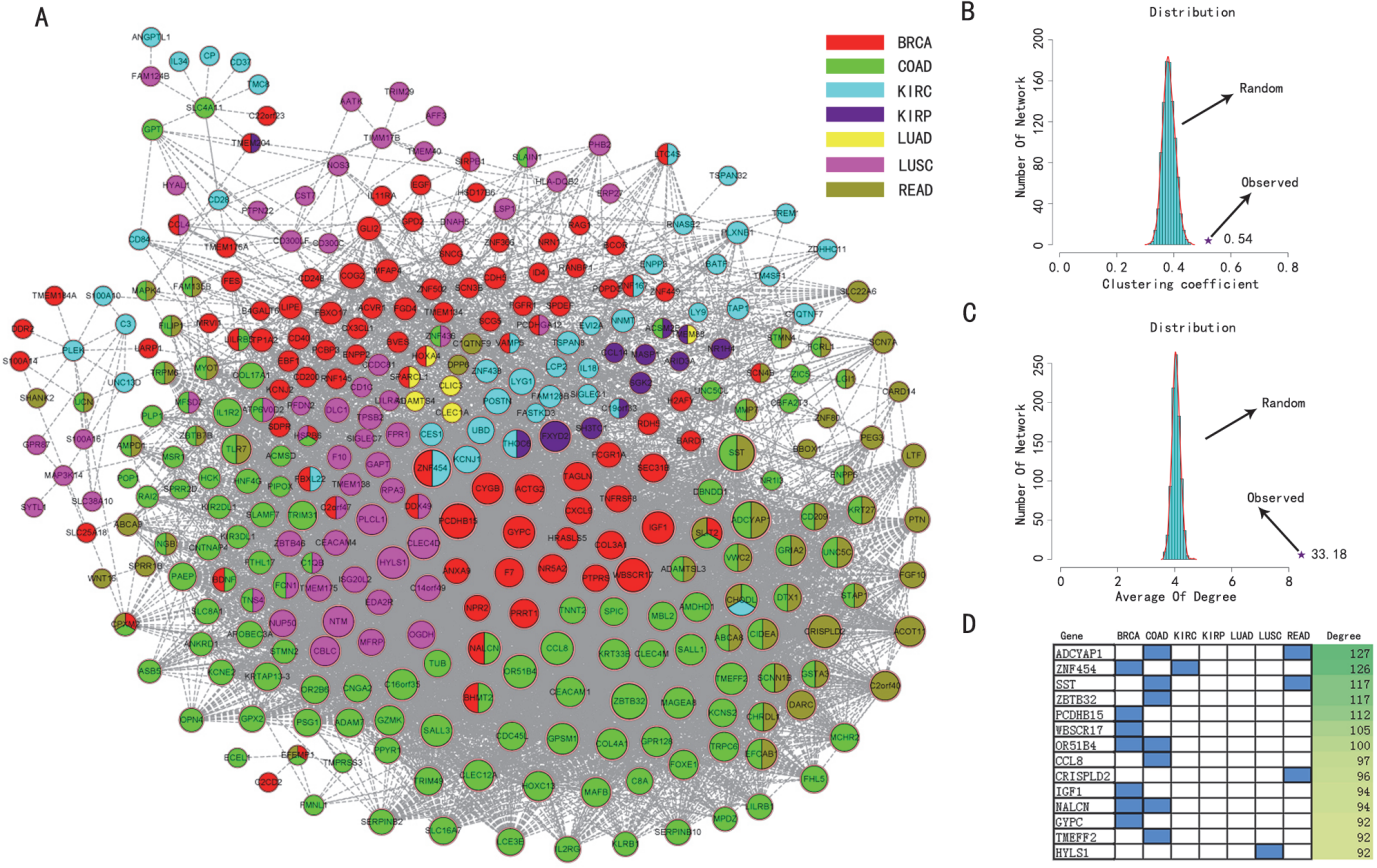
The DMCN of differentially methylated genes. (A) The color stood for different cancers. The size of the nodes stood for the degree of the genes. The line stood for the correlation between two genes. (B) Distribution of clustering coefficient. Y axis was the distribution of random networks; x axis was the clustering coefficient. The star was the value we calculated (C) Distribution of degree. (D) Hub genes and degrees. Blue bar stood for which cancers the differential genes located in.

In order to investigate the relationship across cancers with DNA methylation medium, we quantified the correlation of DMGs based on their methylation patterns across cancers. A differential DNA methylation correlation network (DMCN) was constructed according to the Pearson correlation coefficients of methylation levels, which was an undirected graph displaying the complex relationship of a great deal of genes and seven cancers. DMCN consisted of 5,492 edges and 331 nodes respectively (Fig. 4A). The intersection between DEGs and DMGs were used as nodes, meanwhile edges were weighted by methylation correlation coefficients between DMGs.

We analyzed DMCN to demonstrate the reliability of our network by two topological structures separately, including clustering coefficient and average of degree. First, we perturbed the data in every genes and calculated the correlation coefficients (p< = 0.01) and averages of degree (1000 times). Then we compared clustering coefficients (0.54) and average of degree (33.18) in DMCN to the random networks (Fig. 4B C). The result showed that our network was more correlated and significant than random networks. Moreover, hub genes were a class of genes with high degree and played an important role as key regulatory features in disease network. The degree was from 1 to 127 and the genes with top 10 degrees were defined as hubs (Fig. 4D). The hub genes included ADCYAP1, ZNF454, ZBTB32, SST, PCDHB15, WBSCR17, OR51B4, CCL8, CRISPLD2, NALCN, IGF1, TMEFF2, HYLS1 and GYPC. From the result, we found that the significant genes were mainly distributed in BRCA and COAD, which was displayed strong correlation between the two cancers [32].

As shown in Fig. 4A, seven colors were used to distinguish the nodes which cancers they belonged to. There were 72 genes showing specific methylation in BRCA, 81 in COAD, 36 in KIRC, 7 in KIRP, 3 in LUAD, 48 in LUSC and 19 in READ in the network. The nodes in network were mainly classified into three categories including triple differential (TRI_D, 5 genes), double differential (DOU_D, 60 genes) and single differential (SIN_D, 266 genes). For example, *SLIT2* of TRI_D was abnormal in BRCA, COAD and READ, which showed negative relationship between DNA methylation and gene expression. *SLIT2* was usually regarded as a tumor suppressor gene and silenced both in colorectal cancer and breast cancer, whose silence was caused by the hypermethylation of its promoter regions and allelic loss [33,34]. Gene *EFEMP1* as a member of fibulin family also was abnormal in BRCA, COAD and READ. This gene was related to the angiogenesis and was described as key element of cancer progression. The down-regulation of it was due to the hypermethylation of promoter. Moreover, the decreased expression of *EFEMP1* seemed to strongly correlate with poor disease-free and overall survival [35,36]. In addition, although *CHODL* was not shown function in previous studies, we found the gene was also aberrant of DNA methylation level in BRCA, COAD and READ and displayed negative regulation between DNA methylation and gene expression. It was suggested that there was a strong relationship among BRCA, CORD and READ. In DOU_D set, there were 31 genes which were differential in COAD and READ. This phenomenon implied the correlation between COAD and READ, which was consistent with the result of hierarchical clustering. About 80% genes were part of SIN_D, displaying widely specific among cancers. Particularly, many cancer-related genes including *CDH5, BVES, CX3CL1, FGFR1, IGF1* and *CD40* were identified in SIN_D. The result suggested that the genes we identified had high association with cancers and played an important role in biological process and in development of cancers.

### Survival analysis for cancer specific genes

**Fig 5:**
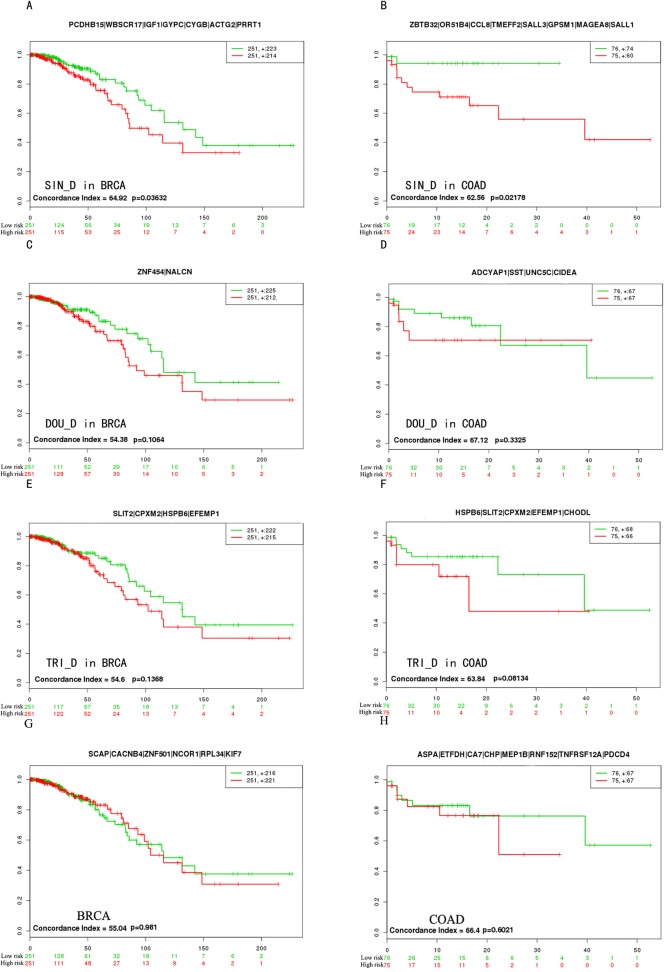
The survival analysis of candidate biomarkers. The “+” stood for the censoring samples. The X axis and Y axis respectively stood for observation time (months) and percent of survival people. Red and Green curves were high-risk group and low-risk group. The biomarkers were on the top of every picture. Concordance Index (CI) and p-value were in the bottom-left insets.

Survival analysis of BRCA and COAD were performed to evaluate the potential roles of candidate biomarkers including all TRI_D genes, the top 10 with high degrees of SIN_D and DOU_D genes, respectively (Table 3). We selected 502 samples of BRCA and 151 samples of COAD which were downloaded from TCGA for the survival analysis. Samples were divided into two groups through the median of PI (prognostic index, high values stood for high risk and low values stood for low risks) and used median risk score as the cutoff. We found SIN_D could separate high-risk group and low-risk group more significantly than DOU_D or TRI_D (Fig. 5). Furthermore the patients with high scores had the shorter survival time. In addition, we used seven genes in BRCA and eight genes in COAD which were differentially expressed but not differentially methylated respectively, to perform the survival analysis as controls. The results of SIN_D in BRCA (p = 0.036) and COAD (p = 0.022) were more significant than their control results (p = 0.981 in BRCA and p = 0.602 in COAD) (Fig. 5 G, H). This result suggested that the combination between DNA methylation and expression could make a better contribution to prognosis than expression only.

**Table 3:** The genes for the survival analysis.

	BRCA	COAD
**SIN_D**	*PCDHB15, WBSCR17, IGF1, GYPC, CYGB, ACTG2, PRRT1*	*ZBTB32, OR51B4, CCL8, TMEFF2, SALL3, GPSM1, MAGEA8, SALL1*
**DOU_D**	*ZNF454, NALCN*	*ADCYAP1, SST, UNC5C, CIDEA*
**TRI_D**	*SLIT2, CPXM2, HSPB6, EFEMP1*	*HSPB6, SLIT2, CPXM2, EFEMP1, CHODL*

### Differentially methylated genes in protein-protein interaction network

**Fig 6:**
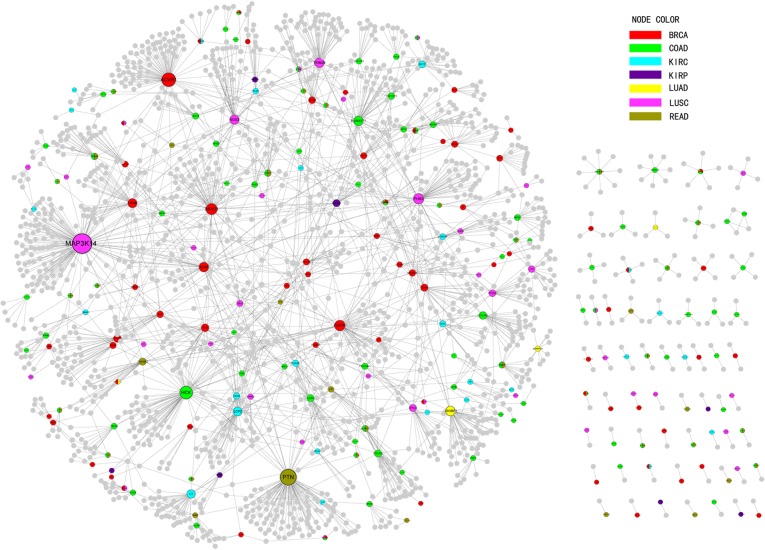
Sub-PPI network of seed genes. The nodes stood for the gene and colors stood for the differential genes in cancers. The grey gene was the biomarkers we forecasted.

Protein-protein interaction networks could be more accurate to describe the relationships between complex elements and more visible to display the constructions of these elements. In order to explore significance of DMGs which were related with cancers, PPI sub-network was built by published PPI network. There were a lot of databases storing the interactions of genes, including the Biomolecular Interaction Network Database (BIND), the Biological General Repository for Interaction Data sets (BioGRID), the Database of Interacting Proteins (DIP), the Human Protein Reference Database (HPRD), IntAct, the Molecular IN Teraction database (MINT), the mammalian PPI database of the Munich Information Center on Protein Sequences (MIPS), PDZBase (a PPI database for PDZ-domains) and Reactome[37–45]. Because many experimental factors might influence the result of PPI network, which led to the repeatable interactions in PPI network, we integrated a high-confidence PPI network based on the above-mentioned databases as our background network [46]. The network we built was composed of 80,980 edges and 13,361 nodes. Therefore we mapped SIN_Ds, DOU_Ds and TRI_Ds as seed genes into the PPI network. The interactions including seed genes were retrieved corresponding to 2,272 edges and 1,892 nodes as a sub-network (Fig. 6). According to the sub-network, there were four genes as hubs ranking in the top 10 degrees, including *MAP3K14, PTN, ACVR1*, and *HCK*, and they were all seed genes. The results suggested that DMGs might play an important role in carcinogenesis. For example, *MAP3K14* (Mitogen-activated protein kinase kinase kinase 14) hypermethylated in LUSC regulated the NF-κB activity pathway and took part in a NF-κB-inducing signaling to receptors of the tumor-necrosis/nerve-growth factor (TNF/NGF) family [47]. In addition, based on the interaction between genes in the sub-network, we identified 127 genes which degrees were more than three as the novel cancer-related genes. The functional enrichment analysis of these 127 genes using DAVID showed that many cancer-associated biological processes and pathways were significant (S6 Table), such as “regulation of programmed cell death”, “regulation of apoptosis”, “positive regulation of programmed cell death”, “positive regulation of transcription” and so on. From the result we realized that the biological processes (BP) were focused on the regulation of apoptosis, programmed cell death and regulation of transcription. On account of previous research, these biological processes were related to the cancers [48,49]. Thus the genes predicted based on the PPI network and abnormal genes might be involved in processes of cancers as potential biomarkers.

Moreover, some researches showed that many pathways associated with cancers from KEGG were significant [50,51]. Our analysis showed that the potential biomarkers obtained in this study were enriched in 11 cancer related pathways, and the most significant pathway was “pathways in cancer (hsa05200)” in which there were 33 genes annotated (FDR = 2.44E-15). This pathway included lots of pathways which were related to cancers such as Wnt signaling pathway, mapk signaling pathway, vega signaling pathway and so on[52,53]. Besides, our biomarkers also were annotated in many other cancer-related pathways, for example, “ErbB signaling pathway”, “Chemokine signaling pathway”, “Natural killer cell mediated cytotoxicity” and “Focal adhesion” [54–56]. The result of annotation indicated that the potential biomarkers participated in many progresses of cancers, and acted as an important role in cancers.

## Discussion

Aberrant DNA methylation on gene promoter regions are usually associated with cancers. For example, aberrant DNA methylation of *SIRT1* is frequently observed in different cancers and played an important role in carcinogenesis [57]. Epigenetic inactivation of *ST6GAL1* is indicated a tumor suppressive role in bladder carcinogenesis[58]. In addition, *ST6GAL1* is also associated with breast cancer. According to the examples above, the features of DNA methylation are reflected cancer-specific and cancer-sharing. It is likely that multiple cancers are placed of origin from the same tissues, resulting in the same DNA methylation patterns. For example, there is a study showing that colon and rectal cancers are difficult to distinguish by DNA methylation level [26]. Therefore, it is useful to analyze the relationship among different cancers through integrating DNA methylation from genome-wide.

In this study, the result of hierarchical clustering and Pearson correlation demonstrated that the DNA methylation level of different cancers were influenced by the anatomical origins, moreover the DNA methylation level had stable DNA methylation signatures in same cancers, which is coincident with previous studies[26]. And the DMGs were mainly enriched in defense response, immune response, cell-cell signaling, cell adhesion and cell killing and other cancer-related KEGG pathways by function annotation analysis, suggesting that the damage of these biological processes activate the proliferation of cancer cells and inhibit protection of individuals [59–64]. Additionally, the hypermethylated genes were mainly enriched in multiple cancer-related KEGG pathways compared to hypomethylated genes, and this finding supported previous study of the focal hypermethylation in tumorigenicity[65].

In DMCN, the topological features indicated that the DMCN followed the characteristic of biological network compared to random network and DMGs might have the similar functions due to acting as the similar roles in caners [66]. The enrichment of hub genes in BRCA and COAD supported that the risk of colon cancer maybe increased because of patients with the history of breast cancers in the previous suggestion [32]. In order to estimate the importance of SIN_D, DOU_D and TRI_D, we performed survival analysis using BRCA and COAD. We found that the cancer-specific genes could share the function in cancers much influentially. The result further demonstrated the potential roles of hub genes in DMCN and the importance of integrated analysis between DNA methylation and gene expression.

Additionally, the result in PPI network indicated the accuracy of our prediction and the potential roles of the novel cancer-related genes in cancers. The result demonstrated that our DMGs could not only predict novel biomarkers accurately but also influence the process of cancers.

Finally we used DMCN, survival analysis, PPI network and many other methods to assess the importance of DMGs for the cancers. The analysis among different cancers showed the potential role of DMGs in the research of cancers. However, more and more high throughput data have been obtained such as Reduced Representation Bisulfite Sequencing (RRBS), Bisulfite Sequencing (BS-seq) and Infinium HumanMethylation450, and our data are on a small scale [67–69]. Therefore, our study will make a contribution to the further understanding of development and progression in cancers through the high throughput data and more and more studies are needed for the integrated analysis of different cancers and improve the prediction of biomarkers in cancers.

## Supporting Information

S1 Table The information of DNA methylation and expression data. (XLS)

S2 Table The list of genes existing in seven cancers. (XLS)

S3 Table The list of differentially methylated genes. (XLS)

S4 Table The list of genes for bidirectional hierarchical clustering analysis. (XLS)

S5 Table The result of differentially methylated genes for DAVID. (XLS)

S6 Table The result of biomarker genes in PPI network for DAVID. (XLS)
